# Role of Dietary Supplements and Probiotics in Modulating Microbiota and Bone Health: The Gut-Bone Axis

**DOI:** 10.3390/cells11040743

**Published:** 2022-02-21

**Authors:** Alessandro de Sire, Roberto de Sire, Claudio Curci, Fabiana Castiglione, Walter Wahli

**Affiliations:** 1 Physical Medicine and Rehabilitation Unit, Department of Medical and Surgical Sciences, University of Catanzaro “Magna Graecia”, 88100 Catanzaro, Italy; 2 Gastroenterology Unit, Department of Clinical Medicine and Surgery, University Federico II of Naples, 80126 Naples, Italy; roberto.desire@libero.it (R.d.S.); fabcasti@unina.it (F.C.); 3 Physical Medicine and Rehabilitation Unit, Department of Neurosciences, ASST Carlo Poma, 46100 Mantova, Italy; claudio.curci@asst-mantova.it; 4 Lee Kong Chian School of Medicine, Nanyang Technological University Singapore, Clinical Sciences Building, Singapore 308232, Singapore; 5 Toxalim Research Center in Food Toxicology (UMR 1331), French National Research Institute for Agriculture, Food, and the Environment (INRAE), F-31300 Toulouse, France; 6 Center for Integrative Genomics, University of Lausanne, Le Génopode, CH-1015 Lausanne, Switzerland

**Keywords:** bone health, osteoporosis, microbiota, gut microbiota, dietary supplements, probiotics

## Abstract

Osteoporosis is characterized by an alteration of bone microstructure with a decreased bone mineral density, leading to the incidence of fragility fractures. Around 200 million people are affected by osteoporosis, representing a major health burden worldwide. Several factors are involved in the pathogenesis of osteoporosis. Today, altered intestinal homeostasis is being investigated as a potential additional risk factor for reduced bone health and, therefore, as a novel potential therapeutic target. The intestinal microflora influences osteoclasts’ activity by regulating the serum levels of IGF-1, while also acting on the intestinal absorption of calcium. It is therefore not surprising that gut dysbiosis impacts bone health. Microbiota alterations affect the OPG/RANKL pathway in osteoclasts, and are correlated with reduced bone strength and quality. In this context, it has been hypothesized that dietary supplements, prebiotics, and probiotics contribute to the intestinal microecological balance that is important for bone health. The aim of the present comprehensive review is to describe the state of the art on the role of dietary supplements and probiotics as therapeutic agents for bone health regulation and osteoporosis, through gut microbiota modulation.

## 1. Introduction

Poor bone health is a relatively silent condition that can develop in both adults and the elderly, inducing bone microstructural alterations and osteoporosis, with a decreased bone mineral density (BMD), leading to fragility fractures [1]. Around 200 million people worldwide are affected by osteoporosis [2]. Fragility fractures, due to low-energy trauma, might necessitate hospitalization and surgical interventions, with detrimental consequences, such as permanent disabilities and an increased risk of death [3]. Osteoporosis is associated with several risk factors—primarily endocrine disorders, medication, immobility, lifestyle, and hereditary genetic factors [4].

Today, altered intestinal homeostasis is being investigated as a potential additional risk factor for reduced bone health, and also as a novel therapeutic target [5]. Indeed, the intestinal microbial populations that live in symbiosis with their human host are collectively defined as the human gut microbiota [6,7], composed of more than 1000 different microorganisms, with the majority particular to each healthy subject, along with a common, stable, and abundant component of *Bacteroidetes*, *Prevotellaceae*, and *Faecalibacterium* species [8,9].

Alterations of the gut microbiota—called dysbiosis—which result in an imbalance between the types of microorganisms present in a person’s natural microflora, can dysregulate metabolic processes, leading to inflammatory bowel diseases [10], diabetes [11], malignancies [12], cardiovascular diseases [13], rheumatoid arthritis, and systemic lupus erythematosus [14].

Microbiota dysbiosis is a multifactorial condition that can be triggered by conditions such as stress, inflammation, and aging. In general, after healing of a diseased state, dysbiosis tends to be corrected naturally [15].

Environmental conditions associated with genetic factors of the host can modify their immunological profile and microbiota composition, which can unbalance the microbial community. For example, in animal models, quantitative trait loci encoding for interferons, interleukin-1 receptor-associated kinase 4 (IRAK-4),and transforming growth factor-beta 3 (TGF-β3) modulate the ratio of *Bacteroidetes*, *Rikenellaceae*, and *Prevotellaceae*, respectively [5,16].

Furthermore, the gut microbiota participates in the bone metabolic signaling pathways through the generation and translocation of metabolites. Bone is impacted by gut microbial dysbiosis that both impairs the intestinal absorption of calcium and dysregulates osteoclasts’ activity via the serum levels of insulin-like growth factor 1 (IGF-1) [17]. Moreover, microbiota alterations also reduce bone strength and quality [18,19], and affect the OPG/RANKL pathway in osteoclasts [20]. Furthermore, dysbiosis in mice also interferes with skeletal muscle mass and physical performance, altering the production of rapsyn and Lrp4—two crucial proteins in the functioning of neuromuscular junctions [21]. Similarly, the antibiotic and antiparasitic drug metronidazole causes gut dysbiosis, inducing skeletal muscle atrophy and impacting muscle chronometabolism [22].

Although gaps exist in the understanding of the pathophysiological mechanisms underpinning the interaction between gut microbiota and bone, it has been hypothesized that dietary supplements, prebiotics (non-digestible dietary fibers stimulating selective bacterial growth), and probiotics (microorganisms that might confer a health benefit to the host) contribute to the intestinal microecological balance, which is important for bone health [23,24,25]. Indeed, dietary supplements and probiotics might play a role in the management of osteoporosis, in conjunction with the already-known medical and lifestyle interventions.

For this purpose, we wrote the present comprehensive review after analyzing both animal and human-based model studies published in the past 20 years. Our survey focuses on the roles that dietary supplements and probiotics play as therapeutic agents for the regulation of bone health and osteoporosis, through gut microbiota modulation.

## 2. The Gut-Bone Axis

On the one hand, the gut microbiota shows variability depending on several conditions, such age, sex, diet, environmental conditions, and diseases [26,27]. On the other hand, bone is also a dynamic organ undergoing constant remodeling, maintaining bone mass and calcium serum level homeostasis [26].

Gut physiology and the gut microbial population are both involved in the regulation of bone metabolism at different levels. Gastrectomy causes a loss of BMD in both animal models and humans [28,29], and impaired gastric acid influences calcium metabolism and uptake [30]. In addition, several neuropeptides secreted by the gastrointestinal system (i.e., glucagon-like peptide-1, glucagon-like peptide-2, and ghrelin) enhance bone formation or inhibit bone resorption, whereas others (i.e., gastric inhibitory polypeptide, gastrin, and serotonin), in contrast, act as pro-osteoporotic factors [31,32]. Bacterial variance and the prevalence of individual microbial species in the gut of the host can alter micronutrients, calcium, and vitamin D absorption, and cause systemic inflammation [33].

**Figure 1:**
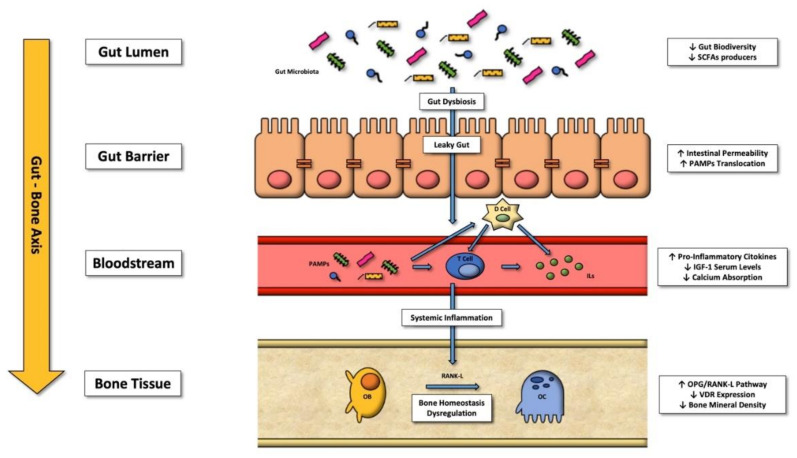
The gut-bone axis: pathways and factors starting from gut dysbiosis that determine bone metabolism alterations favoring osteoclasts. Abbreviations—SCFAs: short-chain fatty acids; PAMPs: pathogen-associated molecular patterns; D Cell: somatostatin-producing cells; T Cell: type of leukocyte that is an essential part of the immune system; OB: osteoblast; OC: osteoclast; RNK-L: receptor activator of nuclear factor-κB ligand; OPG: osteoprotegerin; VDR: vitamin D receptor; IGF-1: insulin-like growth factor one.

The relationship between the gut-bone axis and immunity is complex; under physiological conditions, the gut microbiota stimulates and develops the host’s innate and adaptive immune systems [34]. By contrast, gut microbiota dysbiosis can provoke inflammatory cytokine overproduction, as shown in animal models. Under dysbiosis, bacterial metabolites are delivered to the liver by the blood circulation and upregulate the hepatic immune response. In parallel, they also alter the bone marrow CD4+ T cells, with a resulting pro-osteoclastic effect [20] (see Figure 1 for further details).

In a recent study involving 181 postmenopausal women, divided into 3 groups—normal BMD, osteopenia, and osteoporosis—fecal microbiota sample analysis revealed that the most prevalent microbial species in these conditions statistically correlated with altered bone metabolism [35]. In particular, *Actinomyces* and *Clostridium* species were more prevalent in subjects with osteoporosis, whereas *Bacteroidetes* and *Firmicutes* were more prevalent in healthy individuals. Interestingly, *Actinomyces* might be involved in the development of osteonecrosis of the jaw, whereas *Firmicutes* contribute to an estrogen analogue conversion, with anabolic effects on bone [36]. Interestingly, bacterial populations vary in osteopenia, primary osteoporosis, or secondary osteoporosis, but the relationship and prevalence between microbial species under these conditions are still debated [37]. To date, several substances—including trimethylamine *N*-oxide, *N*-acetyl-mannosamine, l-threonate, l-lysine, and short-chain fatty acids (SCFAs)—have been shown to modulate the gut microbiota, with a consequent impact on bone [37,38]. Therefore, the potential therapeutic role of dietary supplements and probiotics is intriguing and worthy of assessment in people affected by osteoporosis; such interventions might alter the gut microbiota composition, promoting bone health [24].

## 3. Effects of Dietary Supplements on the Gut Microbiota and Bone Health

Diet is the main determinant of the types and proportions of microorganisms in the gut microbiota, influencing bone health and wellness [39]. For example, a high-fat diet reduces microbiota biodiversity in mice, while a highly sweetened diet causes glucose intolerance [40]. From a nutritional point of view, the Mediterranean diet provides fibers, fermented dairy products, and polyphenols, which have beneficial effects on the gut microbiota in humans, reducing the *Firmicutes* and *Bacteroidetes* ratios and enhancing the levels of SCFAs [41], Together, these effects promote bone health and reduce fracture risk [42,43,44,45].

Compared to the known beneficial effects of the Mediterranean diet, it is still uncertain whether supplementation with prebiotics, proteins, peptides, amino acids, and micronutrients might modulate the gut microbiota composition to induce beneficial effects on bone health [46]. Current knowledge on this subject is summarized and discussed below.

### 3.1. Roles of Prebiotics

Prebiotics are non-digestible food ingredients that stimulate the growth and/or activity of bacteria colonizing the large bowel by acting as substrate for them [47]. In most cases, they are products of the enzymatic conversion of sugars, such as galactooligosaccharides (GOSs), fructooligosaccharides (FOSs), inulin, digestion-resistant starch, xylooligosaccharides (XOSs), and lactulose.

GOSs are composed of a chain of galactose (typically 2–8 units) with a terminal glucose, which fosters the growth of *Bifidobacteria* and *Lactobacillus* [47], which are known to increase the quantity and activity of osteoblasts [48,49]. Moreover, GOSs increase the intestinal absorption of calcium and magnesium, along with bone mineralization in animal models [50]. In postmenopausal women, their consumption leads to increased calcium absorption and stable urinary calcium excretion [51]. Lastly, in a double-blind trial, adolescent girls receiving 5 g of GOSs twice a day for 3 weeks increased both calcium absorption in the lower gut and fecal *Bifidobacteria* [52].

FOSs are composed of 3–10 fructose units, with the terminal fructose linked to a glucose residue. FOSs show similar effects to those of GOSs concerning the growth stimulation of *Bifidobacteria* [53], associated with an increase in colon concentrations of butyrate—an SCFA that, along with propionate, regulates the gut microbiota, in particular with respect to its action on bone metabolism. FOS ingestion has been correlated with increased bone strength, mineralization, and reduced bone resorption in animal models [54,55,56]. In another, more recent animal study, FOSs increased the maximum amount of bone mass—the so-called “peak bone mass”—and rised the serum level of butyrate in ovariectomized rats affected by bone loss caused by estrogen deficiency [57]. In humans, FOS supplementation (3.6 g/day for 12 months) coupled with calcium supplementation reduced the serum levels of bone turnover markers in postmenopausal women, with no effect on BMD [58].

Resistant starches are a subgroup of dietary fibers that reach the colon undigested, as the small intestine is not able to alter their composition. These fibers are utilized as substrates by the microbiota in the colon, and their fermentation produces SCFAs [59]; they also promote soya isoflavone production, and increase the ratio of *Bifidobacteria*, *Lactobacillus* species, and *Bacteroides* [60,61]. These starches reduce bone loss in ovariectomized mice [61], decrease inflammation, and interfere with the RANKL/OPG pathway [62]. 

XOSs are prebiotics composed of 2–7 xylose molecules fostering *Bifidobacteria* over *Lactobacillus* [63]; in mice, they increase calcium uptake by upregulating the expression of transient receptor potential vanillin receptor 6 (TRPV6) and Na^+^/Ca^2+^ exchanger in the duodenum, and increase BMD [64]; they also reduce bone loss in high-fat-diet insulin-resistant mice [65], and decrease inflammation markers.

Finally, oral supplementation of lactulose (20 gr/kg per 6 weeks) inhibits osteoclastogenesis, reduces bone resorption, and prevents ovariectomy-induced bone loss in mice [66], while increasing calcium and magnesium absorption in both men and postmenopausal women [67,68].

The prebiotics have in common the ability to be converted to SCFAs—including acetate, propionate, and butyrate—by the gut microbiota, increasing both their intestinal and serum levels, and lowering the intestinal pH. In an acidic environment, most minerals—such as magnesium and calcium—become more soluble; hence, their absorption is increased [69,70,71]. Butyrate also acts as a growth factor for enterocytes and colonocytes [72].

Moreover, SCFAs generated from prebiotics regulate the number and function of regulatory T cells in the colon, thereby controlling inflammation [73], and modulate the synthesis of IGF-1 involved in bone remodeling [74]. Therefore, supplementation with saccharides might have beneficial effects on bone metabolism, although further studies are warranted to generalize these findings (see Table 1 for further details).

### 3.2. Proteins, Peptides, and Amino Acids

Intake of dietary proteins—in particular those from dairy products—has been linked with bone size, bone mass, and strength [75]. Some peptides contained in these products solubilize calcium and promote its absorption by the intestine [75]. Furthermore, the production of hepatic IGF-1 is stimulated by aromatic amino acids that are abundant in fermented dairy products, and this growth factor regulates bone growth [76]. Indeed, a recent study suggested that consumption of fermented dairy products might slow the age-related cortical bone loss in non-weight-bearing bone sites, independent of total energy, calcium, or protein intake, in a cohort of healthy postmenopausal women [77]. Furthermore, ingestion of fermented dairy products was associated with increased bone mass and reduced risk of fracture in the elderly [78,79], partly due to its content in lactulose-derived products which, together with probiotics, reduce the serum levels of parathyroid hormone (PTH), promoting bone resorption and, consequently, bone resorption markers [80].

Among dairy products, kefir is a complex fermented product generated through the symbiotic fermentation of milk by lactic acid bacteria and yeasts encased in a protein-and-polysaccharide matrix. Kefir supplementation for 8 weeks in ovariectomized mice caused increased BMD, increased the number and thickness of bone trabeculae, increased bone volume, and promoted better mechanical properties and fracture toughness [81,82]. In the same study, effects on the variability of gut microbiota were also shown, with a normalization of fecal levels of *Alloprevotella*, *Anaerostipes*, *Parasutterella*, *Romboutsia*, *Ruminococcus*, and *Streptococcus* species that were all more abundant compared to the sham group [82]. Bacterial species might hydrolyze proteins in the intestine, although this process can be modified by diet [83]. A higher gut bacterial production of tyrosine and tryptophan, resulting in elevated levels of these amino acids, might increase osteoporosis [84], while reduced gut degradation of the branched-chain amino acids valine, leucine, and isoleucine—and, consequently, higher serum levels of them—was associated with the prevalence of specific gut microbiota species [85], and was inversely correlated with the occurrence of osteoporosis [86,87] (see Table 2 for further details).

### 3.3. Micronutrients

Several micronutrients other than calcium might play a role in bone mineralization and growth [88], and in some cases their levels are strictly linked to the gut microbiota composition [89].

Selenium deficiency can affect bone growth during development [90], and low serum values of this micronutrient have been correlated with low BMD in the elderly [91]. Supplementation with selenium modifies the gut microbiota composition, promoting a dose-dependent increase in bacterial variety [92]. Moreover, zinc is a micronutrient that participates in bone metabolism, as it affects collagen synthesis and alkaline phosphatase activity [93]; its deficiency is correlated with lower BMD in postmenopausal women [94]. In animal models, zinc supplementation increased the number of *Lactobacillus*, while its deficiency caused a reduced ratio of Firmicutes, with lower SCFA production [95,96]. Lastly, both iron deficit and excess were linked to osteoporosis [97]. Low iron levels increased the expression of the fibroblast growth factor 23 (*FGF23*) gene [98]. Transgenic mice overexpressing *FGF23* in osteoblasts presented a rachitic bone phenotype with a bone mineralization deficit, and showed increased levels of osteoblast markers, but the numbers of osteoclasts were slightly reduced or unchanged [99]. Adamts1 was highly induced in rachitic bones of FGF23 transgenic mice, and participated in the degradation of non-mineralized bone matrix collagen [99]. Conversely, a blockade of FGF23 signaling improved the rachitic bone phenotypes [100]. Unsurprisingly, therefore, serum iron levels are linked to BMD, and an adequate intake of this micronutrient is recommended [101]. Iron intake modifies the gut microbiota, increasing *Bacteroidetes* and *Bifidobacterium* [102,103], when introduced with the diet. When supplemented at high doses, iron decreases *Bifidobacterium*, resulting in increased bacterial pathogenicity [104,105]. Therefore, among the several micronutrients with beneficial bone effects, selenium, zinc, and iron can impact the gut microbiota, strengthening the interaction between bone and gut microbial populations (see Table 3 for further details).

## 4. Impact of Probiotics on the Gut Microbiota

Probiotics are defined as “live micro-organisms which, when given in adequate amounts, confer a health benefit to the host” [106], and are made available via yogurts, milk-based foods, powders, capsules, or oral solutions. Their main characteristics include acid and bile tolerance that confers survival in the gastrointestinal tract (GIT), phenotypic and genotypic stability, adhesion to the mucosal surface, antibiotic resistance, and production of antimicrobial factors able to inhibit known pathogens [107]. The more frequently administered probiotics include *Lactobacillus*, *Bifidobacterium*, *Escherichia*, *Enterococcus*, and *Bacillus subtilis*—but also yeasts such as *Saccharomyces*—at a concentration of approximately of 10^7^ to 10^8^ cells per gram of probiotic supplement, with a serving size of 100 to 200 mg [108]. Given the mechanism of action of probiotics, most studies have failed to report any impact on gut microbiota composition. In fact, the effects of probiotics do not lie in their ability to directly restore an altered gut microbiota composition, but rather in contributing genes and metabolites that directly influence epithelial and immune cells, promoting gut health through regulation of the intestinal pH and permeability, along with resistance to colonization by unwanted bacteria [109,110,111,112]. 

The beneficial effects of the administration of probiotics on bone tissue are widely recognized, such that they have been named by some authors “the new calcium and vitamin D” for bone health [108]. Several studies have demonstrated that probiotics (i.e., *L. reuteri*, *L. paracasei*, and *L. helveticus*) prevent bone loss in the ovariectomy-induced postmenopausal mouse model [113,114,115,116]. *Lactobacillus reuteri* administration prevents type-1-diabetes-induced osteoporosis by inhibiting TNFα-mediated suppression of Wnt10b; it also enhances bone density via increased osteoblast activity and decreased bone marrow adiposity [117]. Similarly, it was documented that *Lactobacillus reuteri* administration ameliorates trabecular bone loss, restoring Wnt10b suppression in the bones of mice with glucocorticoid-induced osteoporosis [118]. Wnt10b is thought to be an endogenous regulator of bone formation; one way whereby it increases osteoblastogenesis is by downregulating the expression of PPAR-γ [119].

A randomized double-blind controlled trial showed that the administration of a multispecies probiotic supplement for six months significantly decreased bone-specific alkaline phosphatase and collagen type 1 crosslinked C-telopeptide levels in postmenopausal women with osteopenia, slowing down the rate of bone turnover [120]. Similarly, another study reported beneficial effects of a novel red clover extract rich in isoflavone aglycones, and probiotics against postmenopausal osteopenia, characterized by an attenuation of estrogen-deficiency-related BMD loss and an improvement of bone turnover in women [121]. Furthermore, in a double-blind placebo-controlled study, 12-month daily administration of *Lactobacillus reuteri* decreased the loss of tibia total volumetric BMD in elderly women [122]. 

Therefore, probiotics might play a key role in bone tissue, through modulation of the gut microbiota, providing a health benefit in frail subjects, although their role still needs to be characterized further (see Table 4 for currently known roles).

## 5. Interventional Microbiota Modulation to Reduce Bone Loss

GIT and bone tissue interact with one another through a complex network modulated by the gut microbiota, in which osteoblasts, osteoclasts, and receptor activator of nuclear factor kappa-Β ligand (RANKL) participate [123]. Recently, several studies have focused on establishing the role of the gut microbiota in the onset of osteoporosis, considering its modulation as a potential therapeutic strategy to reduce bone loss [124,125,126]. For instance, restoring gut microbiota eubiosis has positive effects on the treatment of dysbiosis-related extraintestinal disorders—for instance, in bones and muscles—such as osteoporosis, osteoarthritis, and sarcopenia [123,124,127,128,129,130,131,132]. In particular, gut microbiota modulation can be achieved primarily through diet, but also with probiotics, prebiotics, synbiotics (mixtures of probiotics and prebiotics that beneficially affect the host), postbiotics (bioactive compounds produced by food-grade microorganisms during fermentation), and obviously also by antibiotics and fecal microbiota transplantation (FMT) [132,133,134,135].

Diet represents the main environmental factor contributing to the interindividual differences in gut microbiota composition in healthy people [134,135,136,137]. A balanced diet is fundamental for human health, to which a well-balanced complex microorganism ecosystem composed of more than 10^14^ bacteria—as well as fungi, viruses, phages, parasites, and archaea, which colonize the GIT—is a major contributor [41]. On the *other* hand, high-fat diets lead to a decrease in the numbers of “good bacteria”, such as Bacteroidetes, and an overgrowth of opportunistic pathogens, able to promote a disruption of the intestinal epithelial tight junctions. This leads to an increase in gut permeability, allowing the translocation of bacterial lipopolysaccharides into the systemic blood circulation (so called “leaky gut syndrome”), promoting systemic inflammation and insulin resistance [138,139]. In contrast, high-fiber diets and fructooligosaccharides are associated with an increased number of *Bifidobacteria* species producing SCFAs, such as acetate, propionate, and butyrate, which enhance glucose uptake and insulin sensitivity [140]. SCFAs also play a role in regulating bone homeostasis by downregulating both osteoclast activity that causes bone resorption and inflammation sustained by T-reg lymphocytes [141]. In this context, the adherence to a Mediterranean diet—rich in fibers, fermented dairy products, and polyphenols—is associated with lower hip fracture risk [142]. In association with diet, physical exercise has an impact on several cortical bone parameters and bone mechanical properties—possibly by modulating the intestinal microbial balance toward a decrease in the Firmicutes/Bacteroidetes ratio and an increase in the number of Actinobacteria phylum members, such as *Bifidobacteriaceae*. Modulating the gut microbiota with probiotics can promote the overgrowth of not only Lactobacillus and *Bifidobacterium* species, but also *Faecalibacterium*, *Akkermansia*, *Ruminococcus*, and *Roseburia* species, leading to a stimulation of SCFA production [143,144,145,146]. Furthermore, prebiotics exert a direct antiadhesive effect against pathogens by interacting with bacterial receptors, thereby inhibiting pathogen colonization [147,148]. Several experimental studies [149,150,151,152], both in animals and in humans, support the hypothesis that prebiotics play a key role in the intestinal absorption of calcium and magnesium by causing a decrease in the cecal pH, which results in an increase in BMD.

**Figure 2:**
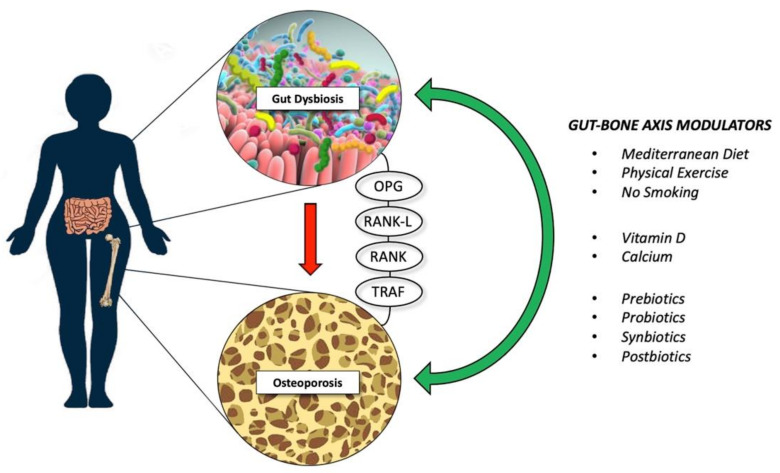
Lifestyle intervention and supplementation that might reduce bone loss; these modulators might exert their therapeutic effects through different known biological pathways, such as OPG, RANK, and TRAF. Abbreviations—OPG: osteoprotegerin; RANK-L: receptor activator of nuclear factor-κB ligand; RANK: receptor activator of nuclear factor κB; TRAF: tumor necrosis factor receptor-associated factor.

In the fast moving “-biotics” field, postbiotics have been defined recently as “bioactive compounds produced by food-grade micro-organisms during a fermentation process” [153]. Postbiotics is an umbrella term that includes metabolites, SCFAs, microbial cell fractions, functional proteins, extracellular polymeric substances (EPSs), cell lysates, teichoic acid, peptidoglycan-derived muropeptides, and pili-type structures, which may be used to promote health by modulating the gut microbiota [153]. In this context, only a few very recent studies [154,155,156] have shown how the administration of postbiotics ameliorates osteopenia and/or osteoporosis. A study of the effects of cell lysates and supernatants of five native probiotic strains (*L. acidophilus*, *L. reuteri*, *L. casei*, *B. longum*, and *B. coagulans*) in ovariectomized rats showed that the *Bacillus*-coagulans-derived-postbiotics had the best impact on bone homeostasis, ameliorating several parameters, such as reduced bone area, bone mineral content, and BMD caused by ovariectomy [153]. Furthermore, a corroborating study [155] using a murine model of postmenopausal-related osteoporosis reported an inhibitory effect of the postbiotic *Lactobacillus curvatus* 38-CS on RANKL-induced osteoclast differentiation and bone loss, by downregulating the TRAF6/NF-κB/MAPKs axis (see Figure 2 for further details).

At this point, it is of interest to discuss the effects and consequences of antibiotics on the gut microbiota. Antibiotics are considered to be deep modulators of the gut microbiota, and the characteristics that can influence their negative or positive impact relate to antibiotic class, dosage, duration, and administration route [156]. On the one hand, their abuse causes several intestinal and extraintestinal disorders associated with gut microbiota impairment—especially *Clostridium difficile* infection. On the other hand, antibiotic therapy can provide a so-called “eubiotic” effect, increasing the abundance of beneficial bacteria, and therefore appears to be a reliable therapeutic strategy for several non-communicable disorders, such as hepatic encephalopathy or irritable bowel syndrome [156]. Antibiotic treatment, by modulating the gut microbiota, can influence bone mass. Animal studies have shown that short-term administration of low doses of antibiotics at weaning results in increased BMD and adiposity, with substantial taxonomic changes in terms of the gut microbiota [157]. Subsequent supporting results showed that a low dose of penicillin delivered from birth to weaning in mice was sufficient to transiently perturb the gut microbiota, inducing sustained effects on body composition with an increase in BMD [158]. In addition, treatment with tetracyclines was found to prevent ovariectomy-induced bone loss, reducing bone resorption modestly and stimulating bone formation substantially [159].

A more recent therapeutic strategy for the modulation of the gut microbiota is the transfer of donor stools to a recipient’s GIT—the so-called fecal microbiota transplants (FMTs), which have gained much recognition recently [160]. Considered to be an established treatment for recurrent *Clostridium difficile* infection, FMT is a potential therapy for inducing remission of mild–moderate ulcerative colitis [161]. Furthermore, its use has been debated in the literature for improving several extraintestinal conditions, including metabolic syndrome, modulation of responses to chemotherapy, eradication of multidrug-resistant organisms, and osteomuscular disorders [161]. In the context of bone disorders, FMT from young to aged rats improved senile osteoporosis in terms of bone volume, trabecular bone volume fraction, trabecular number, and trabecular thickness by restoring gut dysbiosis at the phylum and family levels [162]. Furthermore, FMT improved leaky gut syndrome in aged rats, upregulating the expression of the tight-junction proteins occludin, claudin, and ZO-1 [162]. Interestingly, transplantation of fecal material from mice treated with the corticosteroid prednisolone into naïve, untreated recipients caused bone loss, supporting a role of the gut microbiota in the development of glucocorticoid-induced osteoporosis [118]. However, further studies are required in order to clarify the emerging role of gut microbiota modulation in reducing bone loss to prevent osteoporosis.

## 6. Future Research Avenues and Perspectives for Osteoporosis Treatment

The current available osteoporosis treatments consist of antiresorptive agents that inhibit osteoclast activity, bone-forming compounds that stimulate osteoblasts, and dual-acting drugs that simultaneously inhibit bone resorption and enhance bone accrual [163,164,165]. In bone homeostasis, the Wnt/β-catenin and OPG/RANKL/RANK signaling pathways play crucial roles in bone anabolism and catabolism, respectively [166]. Antiresorptive treatments comprise bisphosphonates and denosumab—an antibody against RANKL [167,168].

Animal studies recently aimed to develop molecules capable of interfering even with the osteoclast–bone matrix proteins (αvβ3 integrin antagonists), acting as bone resorption inhibitors [169,170]. Furthermore, the anabolic bone accrual process can be stimulated by parathyroid hormone analogues (amino acids 1–34) [171]. Moreover, romosozumab is a novel antibody that inhibits sclerostin and, hence, interferes with the sclerostin/Wnt signaling pathway, and might have both anabolic and antiresorptive effects on bone [172]. In the same pathway, frizzled-related proteins (FRPs) are considered to be novel therapeutic targets, and miRNA-based therapies targeting FRPs are under development to prevent and treat osteoporosis [173].

The gut microbiota interferes to some extent with the Wnt signaling pathway, in particular promoting the renewal of hematopoietic stem cells [174]; moreover, it also participates in the OPG pathway and inhibits RANKL [175], possibly similarly to denosumab, but its mechanism is not fully understood. As discussed above, the gut microbiota enhances the intestinal absorption of calcium; interestingly, it might regulate bone metabolism through a calcium-dependent interaction with the Wnt/β-catenin signaling pathway, as demonstrated in animal models [176].

To date, knowledge on gut microbial interaction with the host—and even more so its potential regulatory role in bone health—remains partial [177]. Furthermore, we are aware that the interpretation of animal-model-based studies requires caution with respect to the extrapolation of results to humans, especially considering the clinical management of osteoporosis.

However, several dietary supplements and probiotics highlighted in this review might modulate bone health by acting on the same biological pathways used by some currently utilized drugs. Hence, it might be suggested that the exploration of combined supplement-drug approaches might help to maximize the anti-osteoporotic effects of these interventions.

Finally, it is worth noting that systemic inflammation and immune system effects are often neglected as therapeutic targets in osteoporosis, even though many natural and synthetic substances exert bone effects by acting on T cells or by decreasing the levels of proinflammatory cytokines.

In this review, we highlighted how gut microbiota modulation is appealing as a therapeutic intervention to counteract bone loss. In this context, dietary supplements, prebiotics, probiotics, postbiotics, and synbiotics should be explored further as useful adjuvant treatments for osteoporosis, especially with regards to targeting the most relevant biochemical and signaling pathways. Future human studies are warranted to confirm the hypotheses discussed, in order to build and provide the right support for the clinical management of the gut–bone axis and sustain bone health.

## Figures and Tables

**Table 1:** Characteristics of studies investigating bone effects and gut microbiota modulation of supplementation with prebiotics.

Authors	Journal	Year	Dietary Supplement	Subjects	Pathways Investigated	Gut Microbiota Modulation	Main Findings
Porwal et al. [55]	*Biomedicine & Pharmacotherapy*	2020	Fructooligosaccharides	Animals	SCFA production	Not investigated	Fructooligosaccharides prevent ovariectomy-induced bone loss
Yan et al. [52]	*Carbohydrate Polymers*	2019	Fructooligosaccharides	Animals	Osteoblast stimulation	Not investigated	Fructooligosaccharides increase bone mineral density and strength
Tanabe et al. [54]	*Journal of Agriculture and Food Chemistry*	2019	Fructooligosaccharides	Animals	↓ Serum CRP; SCFA production	↑ *Lactobacillus* ↑ *Bacteroides* ↓ *Clostridium*	Fructooligosaccharides reduce bone resorption and systemic inflammation
Slevin et al. [56]	*The Journal of Nutrition*	2014	Fructooligosaccharides	Humans	Reduction in the number of bone turnover markers	Not investigated	Fructooligosaccharides reduce postmenopausal bone loss
Mathey et al. [53]	*Calcified Tissue International*	2004	Fructooligosaccharides	Animals	SCFA production	Not investigated	Fructooligosaccharides increase bone mineral density and strength
Whisner et al. [50]	*British Journal of Nutrition*	2013	Galactooligosaccharides	Humans	SCFA production	↑ *Bifidobacteria*	Galactooligosaccharides increase calcium absorption
Weaver et al. [48]	*Journal of Agricultural and Food Chemistry*	2011	Galactooligosaccharides	Animals	SCFA production	↑ *Bifidobacteria*	Galactooligosaccharides increase calcium absorption
Van den Heuvel et al. [49]	*The Journal of Nutrition*	2000	Galactooligosaccharides	Humans	SCFA production	Not investigated	Galactooligosaccharides increase calcium absorption
Karakan et al. [65]	*Frontiers in Nutrition*	2021	Lactulose	Humans	SCFA production	↑ *Bifidobacteria* ↑ *Lactobacillus* ↓ *Clostridium*	Lactulose increases calcium absorption
Chen et al. [64]	*Aging and Disease*	2020	Lactulose	Animals	↓ TNFα ↓ RANKL ↓ IL-17 ↑ IL-10	↓ *Firmicutes* ↑ *Bacteroides*	Lactulose inhibits osteoclastogenesis and bone resorption
Seki et al. [66]	*Journal of* *Nutritional* *Science and* *Vitaminology*	2007	Lactulose	Humans	Not investigated	Not investigated	Lactulose increases calcium and magnesium absorption
Tousen et al. [60]	*Nutrients*	2019	Resistant starch	Animals	↓ IL-7R mRNA↑ IL-10 mRNA↓ RANKL	↑ *Bifidobacteria*	Resistant starch prevents post-ovariectomy bone loss
Tousen et al. [59]	*British Journal of Nutrition*	2016	Resistant starch	Animals	↓ IL-7R mRNA	↑ *Bifidobacteria*	Resistant starch prevents post ovariectomy bone loss
Tousen et al. [58]	*Metabolism*	2011	Resistant starch	Animals	Increased isoflavone availability and estrogenic activities	↑ *Bifidobacteria*	Resistant starch prevents post-ovariectomy bone loss
Gao et al. [62]	*Nutrients*	2020	Xylooligosaccharides	Animals	↑ Na^+^/Ca^2+^ exchanger 1 ↑ TRPV6	Not investigated	Xylooligosaccharides increase calcium absorption
Eaimworawuthikul et al. [63]	*European Journal of* *Nutrition*	2020	Xylooligosaccharides	Animals	Osteoclast inhibition	Not investigated	Xylooligosaccharides reduce bone resorption in systemic inflammation

Abbreviations—SCFA: short-chain fatty acid; CRP = C-reactive protein; IL-7R: interleukin-7 receptor; IL-10: interleukin-10; RANKL: receptor activator of nuclear factor kappa-Β ligand; TRPV6: transient receptor potential vanilloid subfamily member 6; TNFα: tumor necrosis factor-alpha; IL-17: interleukin-17; ↓ = lower; ↑ = higher.

**Table 2:** Characteristics of studies investigating bone effects and gut microbiota modulation of supplementation with proteins, peptides, and amino acids.

Authors	Journal	Year	Dietary Supplement	Subjects	Pathways Investigated	Gut Microbiota Modulation	Main Findings
Ling et al. [86]	*The Journal of Clinical* *Endocrinology and Metabolism*	2021	Amino acids	Humans	Not determined	↓ *Actinobacillus*, ↓ *Blautia*, ↓ *Oscillospira*	Valine, leucine, and isoleucine serum levels are inversely related to the occurrence of osteoporosis
Jennings et al. [85]	*Journal of Bone and Mineral* *Research*	2016	Amino acids	Humans	↑ IGF1	Not investigated	Alanine, arginine, glutamic acid, leucine, lysine, and proline increase BMD
Dawson-Hugler et al. [75]	*Osteoporosis* *International*	2007	Amino acids	Humans	↑ IGF1	Not investigated	Phenylalanine and histidine increase calcium absorption
Liu et al. [74]	*Food & Function*	2018	Casein Phosphopeptides	Animals	↑ TRPV6	Not investigated	Casein phosphopeptides increase calcium absorption and prevent bone resorption
Ong et al. [78]	*Advances in Nutrition*	2020	Fermented dairy products	Humans	↓ TNF-α ↓ IL-6 ↓ RANKL	Not investigated	Fermented dairy products might reduce hip fracture risk
Biver et al. [76]	*Osteoporosis International*	2018	Fermented dairyproducts	Humans	Action on calcium balance and decrease in secondary hyper parathyroidism.	Not investigated	Fermented dairy products attenuate postmenopausal bone loss
Laird et al. [77]	*Osteoporosis International*	2017	Fermented dairy products	Humans	Modulation of osteoclast numbers and activity	Not investigated	Fermented dairy products increase bone mineral density
Tu et al. [81]	*Nutrients*	2020	Kefir peptides	Animals	↓ TNF-α ↓ RANKL	↑ *Alloprevotella*, ↑ *Anaerostipes*, ↑ *Parasutterella*, ↑ *Romboutsia*, ↑ *Ruminococcus*, ↑ *Streptococcus*	Kefir peptides prevent ovariectomy-induced bone loss
Chen et al. [80]	*Osteoporosis International*	2014	Kefir peptides	Animals	↑TRPV6	Not investigated	Kefir peptides prevent ovariectomy-induced bone loss

Abbreviations—BMD: bone mineral density; RANKL: receptor activator of nuclear factor kappa-Β ligand; TRPV6: transient receptor potential vanilloid subfamily member 6; TNFα: tumor necrosis factor-alpha; IL-6: interleukin-6; IGF1: insulin growth factor-1; ↓ = lower; ↑ = higher.

**Table 3:** Characteristics of studies investigating bone effects and gut microbiota modulation of supplementation with micronutrients.

Authors	Journal	Year	Dietary Supplement	Subjects	Pathways Investigated	Gut Microbiota Modulation	Main Findings
Qasem et al. [100]	*BMC* *Pediatrics*	2017	Iron	Humans	Lowering inflammation	*↑ Bifidobacteria* *↑ Bacteroides*	Favorable effects of iron on bone might be mediated by the gut microbiome
Wang et al. [89]	*BMC* *Musculoskeletal Disorders*	2019	Selenium	Humans	Not determined	*↓ Parabacteroides* *↓ Firmicutes*	Selenium deficiency is correlated with a higher prevalence of osteoporosis
Reed et al. [94]	*Nutrients*	2015	Zinc	Animals	SCFA production Lowering of inflammation	*↑ Lactobacillus*	Favorable effects of zinc on bone might be mediated by the gut microbiome

Abbreviations—SCFA: short chain fatty acid; ↓ = lower; ↑ = higher.

**Table 4:** Characteristics of the studies investigating bone effects and gut microbiota modulation of supplementation with probiotics.

Authors	Journal	Year	Dietary Supplement	Subjects	Pathways Investigated	Gut Microbiota Modulation	Main Findings
Narva et al. [113]	*Annals of* *Nutrition and Metabolism*	2007	*Lactobacillus* *helveticus*	Animals	Increasing bone formation	Not investigated	*Lactobacillus* *helveticus* prevents ovariectomy-induced bone loss
Ohlsson et al. [114]	*PLoS* *ONE*	2014	*Lactobacillus* *paracasei*	Animals	↓ TNF-α ↓ IL-1β ↑ OPG	Not investigated	*Lactobacillus* *paracasei* prevents ovariectomy-induced bone loss
Schepper et al. [46]	*The Journal of Bone and Mineral* *Research*	2020	*Lactobacillus* *reuteri*	Animals	↑ Wnt10b	↓ *Clostridium*	*Lactobacillus reuteri* prevents glucocorticoid-induced bone loss
Nilsson et al. [120]	*Journal of* *Internal* *Medicine*	2018	*Lactobacillus* *reuteri*	Humans	Not determined	Not investigated	*Lactobacillus reuteri* prevents bone loss
Zhang et al. [115]	*Endocrinology*	2015	*Lactobacillus* *reuteri*	Animals	↓ TNF-α	Not investigated	*Lactobacillus reuteri* prevents type-1-diabetes-induced bone loss
Britton et al. [111]	*Journal of* *Cellular Physiology*	2014	*Lactobacillus* *reuteri*	Animals	↓ Trap5 ↓ RANKL ↑ CD4^+^ T-lymphocytes	Promoting gut microbiota diversity	*Lactobacillus reuteri* prevents Ovariectomy-induced bone loss
Jafarnejad et al. [118]	*Journal of the American* *Nutrition* *Association*	2017	Multispecies probiotic	Humans	↓ PTH ↓ TNF-α	Not investigated	Multispecies probiotic reduces bone turnover

Abbreviations—RANKL: receptor activator of nuclear factor kappa-Β ligand; TNFα: tumor necrosis factor-alpha; Trap5: serum band 5 tartrate-resistant acid phosphatase; CD4^+^ T^−^: cluster differentiation 4 positive cT helper cells; IL-1β: interleukin-1 beta; OPG: osteoprotegerin; Wnt10b: Wnt family member 10B; PTH: parathyroid hormone; ↓ = lower; ↑ = higher.

## Data Availability

Not applicable.
